# Developing mHealth Remote Monitoring Technology for Attention Deficit Hyperactivity Disorder: A Qualitative Study Eliciting User Priorities and Needs

**DOI:** 10.2196/mhealth.5009

**Published:** 2016-03-23

**Authors:** Lucy Simons, Althea Z Valentine, Caroline J Falconer, Madeleine Groom, David Daley, Michael P Craven, Zoe Young, Charlotte Hall, Chris Hollis

**Affiliations:** ^1^ NIHR MindTech Healthcare Technology Co-operative University of Nottingham Nottingham United Kingdom; ^2^ Division of Psychiatry and Applied Psychology School of Medicine University of Nottingham Nottingham United Kingdom; ^3^ NIHR Collaboration for Leadership and Applied Health Research and Care East Midlands University of Nottingham Nottingham United Kingdom

**Keywords:** attention deficit hyperactivity disorder, mHealth, eMental Health, remote monitoring technology, mental health services, qualitative methods, feasibility testing, user requirements

## Abstract

**Background:**

Guidelines in the United Kingdom recommend that medication titration for attention deficit hyperactivity disorder (ADHD) should be completed within 4-6 weeks and include regular reviews. However, most clinicians think that weekly clinic contact is infeasible, and audits have shown that this timeline is rarely achieved. Thus, a more effective monitoring and review system is needed; remote monitoring technology (RMT) may be one way to improve current practice. However, little is known about whether patients with ADHD, their families, and clinicians would be interested in using RMT.

**Objective:**

To explore patients’, parents’, and health care professionals’ views and attitudes toward using digital technology for remote monitoring during titration for ADHD.

**Methods:**

This was a qualitative study, and data were collected through 11 focus groups with adults and young people with ADHD, parents of children with ADHD, and health care professionals (N=59).

**Results:**

All participant groups were positive about using RMT in the treatment of ADHD, but they were also aware of barriers to its use, especially around access to technology and integrating RMT into clinical care. They identified that RMT had the most potential for use in the ongoing management and support of ADHD, rather than during the distinct titration period. Participants identified features of RMT that could improve the quality of consultations and support greater self-management.

**Conclusions:**

RMT has the potential to augment support and care for ADHD, but it needs to go beyond the titration period and offer more to patients and families than monitoring through outcome measures. Developing and evaluating an mHealth app that incorporates the key features identified by end users is required.

##  Introduction

Attention deficit hyperactivity disorder (ADHD) is a neurodevelopmental syndrome characterized by 3 core behaviors—inattention, hyperactivity, and impulsivity—and affects approximately 5% of school-aged children [1]. Left unmanaged, ADHD can result in impairments in multiple domains, including academic performance, productivity, and social adjustment, and can lead to an increased risk of conduct and personality disorders or substance misuse [2]. Medication is the most common treatment, with psychosocial interventions, such as behavior training and parent training, being the main alternatives [3]. Using digital technologies for eMental Health (ie, the use of information and communication technologies (ICT) to support and improve mental health, including the use of online resources, social media, and mobile phone apps [4]) and mHealth (ie, mobile health [5]), has the potential to transform the delivery of mental health care by connecting patients, services, and health data in new ways [6]. Certain features of digital technologies target specific deficiencies for people with ADHD, such as automated reminders and task scheduling to support organizational skills and immediate access to avoid delay and waiting. They also offer the potential to increase access to resource-intensive, and therefore scarce, psychosocial interventions.

Developments of eMental Health for ADHD include digital and Web-based psychometric tools [7]; behavioral interventions [8]; cognitive and biofeedback training packages (some with game-like features) [3,9]; and computerized cognitive assessments [10]. While there is some evidence emerging about these approaches, they remain largely experimental, and have limited alignment to clinical practice [3,9]. For example, the delivery of synchronous and asynchronous behavioral interventions for ADHD with patients and parents has been found to be acceptable, feasible, and effective [8]. Similarly, in child and adolescent mental health services (CAMHS), recent research has shown that eMeasures (ie, outcome measures delivered electronically) are perceived positively by patients and clinicians, and tend to have significantly higher completion rates than do the standard approaches [7,11].

Internet availability and mobile devices are now within the reach of most of the population, enabling new systems of health monitoring to be considered without the previous problem of excluding large sections of the population. In the United Kingdom, 85% of all adults have household access to the Internet, and this rises to 94% among 16-24 year olds; furthermore, mobile Internet appears to have the fastest growing audience (an increase of 11% from 27.2 million unique users in March 2103 to 30.2 million by March 2014) [12]. By March 2015, 66% of all adults owned a mobile phone; among younger age groups, there was nigh-universal ownership (87% for 25-34 years; 90% for 16-24 years) [13]. The mobile phone apps for ADHD currently available through commercial app stores are primarily tools for self-testing and information and management strategies, task and scheduling aids, and brain training games (according to a review of the Google Play and iTunes stores performed in August 2015). While most apps target patients and provide information and non-clinical advice, a small number target clinicians to provide support for treatment decisions. However, most apps have been developed outside of clinical environments and few are supported by an evidence base [14]. Neither is it clear whether potential end-users have been involved in the design and development of these tools. Given these limitations in this and other health areas, serious concerns have been raised about the safety, usability, and effectiveness of unregulated health apps [15].

In the United Kingdom, an identified unmet need in ADHD treatment is the delay in reaching the optimum dose for patients commencing medication (personal communication, unpublished audit, Hall, 2015). During initial medication titration, the National Institute for Health and Care Excellence (NICE) ADHD Guideline [1] recommends that progress be reviewed regularly, such as by weekly telephone contact and at each dose change, and that the entire process should occur over 4-6 weeks [1]. However, weekly contact during titration is not viewed as feasible by most clinicians in the UK National Health Service (NHS) because of time and resource constraints (personal communication (unpublished audit), Hall, 2015). Evidence from the landmark Multimodal Treatment Study of Children with ADHD in the United States [16] highlighted the importance of high quality medication management in ADHD, including carefully monitoring individual dose titration with regular follow-up, in facilitating better outcomes for patients. Therefore, a more effective system is needed to ensure that patients are monitored closely but without increasing the strain on clinic resources.

Remote monitoring technology (RMT) is a means of collecting physiological or health-related data from individuals passively or by their active input on an electronic device, such as a mobile phone, and relaying these data over an internet or phone connection to a remote server. Given the ubiquity of mobile device use and Internet availability in the population, the conditions for using RMT as an adjunct to traditional models of service delivery appear better than ever. Using RMT offers a potential solution to delays in titration currently experienced in the NHS. However, little is known about whether patients and health care professionals in the NHS context would support the introduction of RMT to aid treatment monitoring for ADHD. Moreover, there is a need to ensure the quality of any RMT products and that patient and families’ needs remain at the center of any technology development and implementation [17]. Therefore, the aim of this study was to explore patients’, parents’, and health care professionals’ views regarding the use of RMT during medication titration for ADHD.

##  Methods

### Design and Context

We conducted an exploratory cross-sectional focus group study with patients, parents, and health professionals. Focus groups were chosen as the preferred method of data collection for this study because they enable an exploration of experiences and views and facilitate discussion between participants on a topic of shared interest [18]. The setting was 4 NHS mental health provider areas in the East Midlands region of England, the United Kingdom. An industrial partner [19] developed an initial prototype RMT system, which was used as a vehicle for exploring participants’ views of this technology.

**Figure 1:**
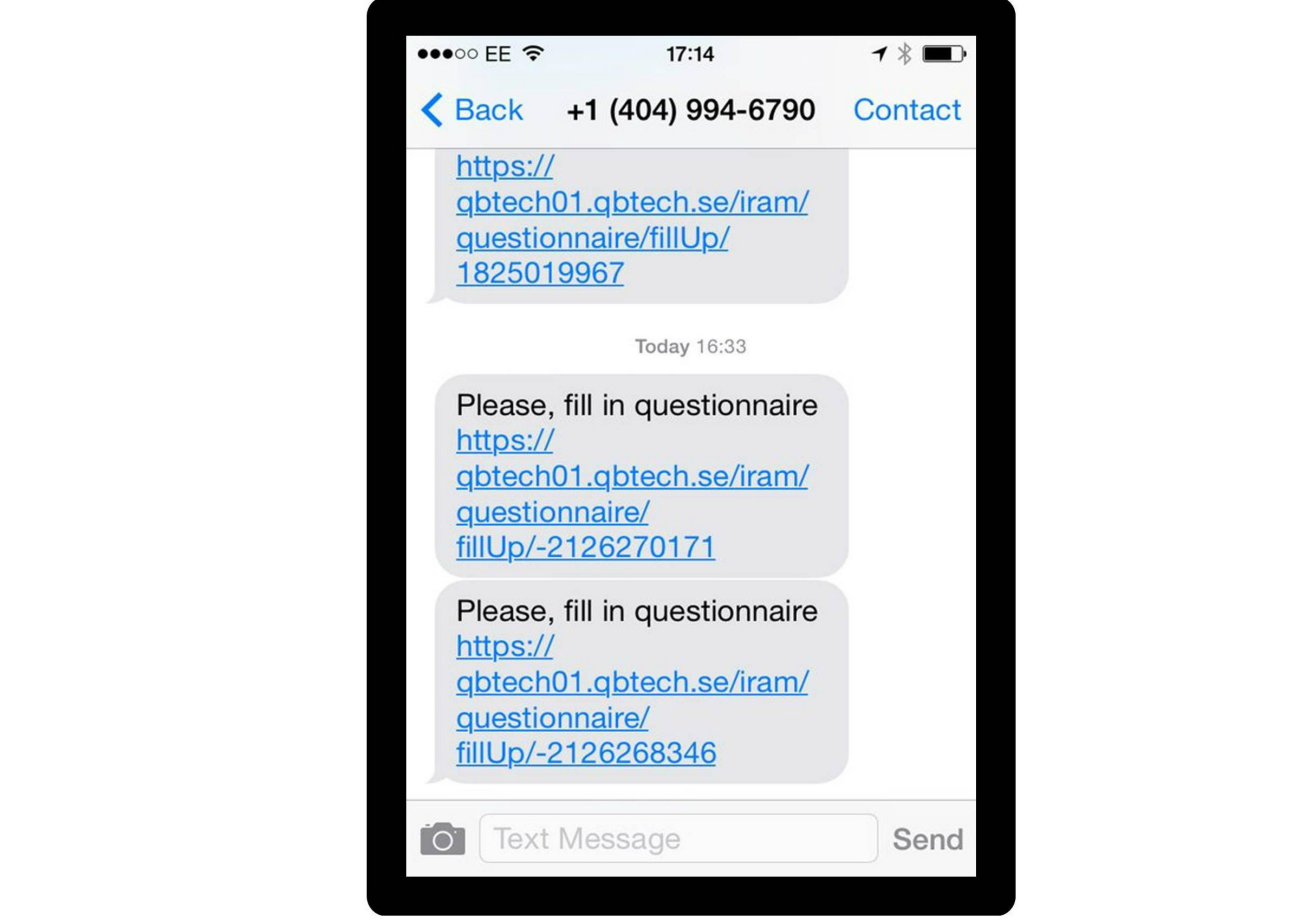
Screenshot of the prototype RMT: Text message received by patient.

**Figure 2:**
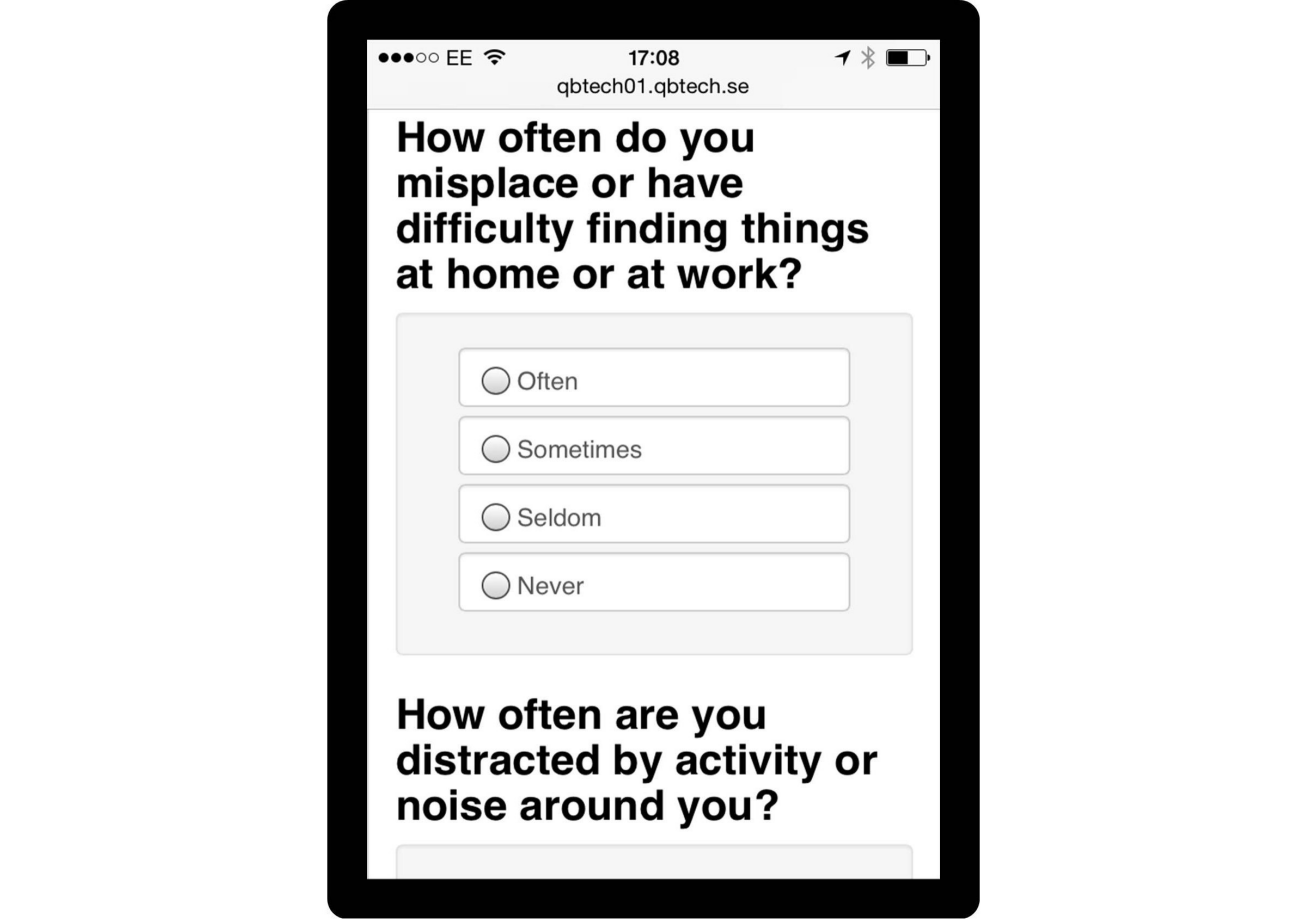
Screenshot of prototype RMT: Example ROM.

**Figure 3:**
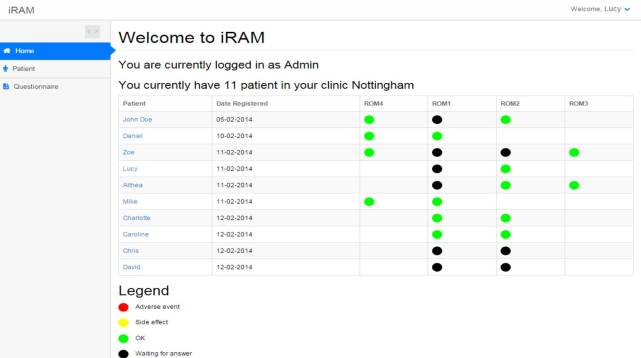
Screenshot of prototype RMT : Clinician dashboard.

The prototype RMT enabled automated text messages to be sent to patients who were invited to complete Web-based versions of routine outcome measures (ROMs) to monitor symptoms and side effects. The system comprised a clinician dashboard where patient details were entered and the specific ROMs required for each individual patient were selected. The dashboard indicated when patients had completed their measures and showed red flags for any issues of concern. To use the RMT patients required a mobile phone to receive text messages and activate the links to the Web-based versions of the ROM. Once completed, the data were relayed back to the system server and the clinician dashboard updated. The primary aim of the prototype was to enable the clinic to receive information about responses to and side effects of medication during the titration period in addition to that obtained from face-to-face appointments. Screenshots of the prototype are displayed in Figures 1-3.

### User Involvement in Study Design

Before undertaking the focus groups, a workshop was held to elicit feedback on the prototype RMT system from potential end-users. It was attended by a young person with ADHD and his parent, 2 adults with ADHD, 1 psychiatrist, 1 psychologist, and 1 medical technology researcher. This workshop identified key factors to explore in more detail in the focus groups. The experience of the workshop suggested that demonstrating the prototype system during the focus groups would risk diverting attention away from the primary research purpose. This was primarily because the “on-boarding” process for the prototype RMT was lengthy—individual patient details needed to be entered into the clinician dashboard and patients needed to both receive and reply to SMS text messages. In the short time we had available in the scheduled focus groups, we determined that this process would dominate the session and limit the time needed for exploring participants’ views and acceptability of the general concept of RMT. Therefore, we decided not to use the prototype RMT in the focus groups, but instead created printed materials from the system to aid in discussion (see Figures 1-3).

### Participants

The study sample comprised 8 young people with ADHD (YP), 11 adults with ADHD (adults), 9 parents of children with ADHD (parents), and 31 health care professionals (HCP) working with people with ADHD (see Table 1). YP, adults, and parents were recruited through 2 routes: NHS ADHD clinics (CAMHS and community pediatrics) or community ADHD support groups.

Inclusion criteria were a self-reported diagnosis of ADHD or having a child with a self-reported diagnosis of ADHD. This was checked by asking participants to confirm where they had received the diagnosis (90% had received it in NHS clinics and 10% in private health care facilities). HCPs were recruited by the local investigators at each NHS provider organization. The criterion for inclusion was employment in a service treating people with ADHD. A purposive sample of HCPs was sought to reflect the range of staff involved in medication management in clinical settings and implementation of any new systems.

### Procedure

Eleven separate focus groups took place in areas with variable relative deprivation including inner cities, post-industrial towns, and semi-rural districts. They were held in locations convenient to the participants (either NHS clinic or support group premises) and took place between June and September 2014. Each group was limited to a single participant type (see Table 1) [18] and we aimed to hold at least 2 focus groups for each participant type. We also made sure that each different mental health care provider was represented by at least 1 HCP focus group to enable an exploration of any differences in practice or attitudes between the different provider organizations [18]. The YP groups lasted for 45 and 50 minutes, while the groups with adults, parents, and HCPs lasted 75-90 minutes.

The study received ethical approval from the UK National Research Ethics Service Committee South Central - Berkshire B (ref 13/SC/0641). Information about the study was sent to all potential participants before the scheduled focus group date. At the beginning of each session, the study was explained to participants and any questions were answered. All adult participants gave their written informed consent to take part. Parents of the YP aged under 16 gave their written informed consent for their children to take part, while the YP gave their verbal assent. YP, adults, and parents were all given a £20 shopping voucher as a reward for their participation. HCPs all took part during their usual working times, so no remuneration was given to them.

Each focus group was facilitated by 2 members of the research team. One member brought domain (insider) knowledge about ADHD (ZY), while the other brought methodological (outsider) knowledge about focus groups (LS or MC). The facilitators sought to remain “background figures” [20] in the groups, guiding the process, rather than leading it. Moreover, the insider (domain) and outsider (methodological) perspectives were combined to ensure the discussions remained focused on the core topic while ensuring that researchers could still seek clarification on assumed knowledge and implied meanings between those with common experiences.

The discussions were guided by a topic schedule covering 4 key areas: using technology for health; medication titration experience/practice; remote monitoring for effects and side effects; and aspirations for using technology to manage ADHD (see Appendix 1). Drawing on best practice advice for focus groups [20], and especially for young people [21], we included different activity-oriented questions in each group (see Table 2). These were designed to enrich the data collected, make it easier to talk about sensitive topics (for example, medication taking), and, importantly for our participants, reduce lapses in attention [22]. Some of these activities were designed to ensure that participants could express their views individually, and thereby avoid inadvertent acquiescence bias to the researchers or censoring by the other participants. Although 2 of the focus groups had only 2 participants, the discussions were similar to those in the larger groups in that they were highly interactive and yielded equally rich data. Participants also completed a short questionnaire on their demographic characteristics and current technology use.

**Table 1:** Number of participants in each focus group by study area

Site	Health care professionals	Adults	Young people	Parents	Total
Site 1	9^a^	4	-	-	13
Site 2	7	7	6	7	27
Site 3	7	-	2	2	11
Site 4	8	-	-	-	8
Total	31	11	8	9	59

^a^ Two focus groups were held with health care professionals at Site 1: 1 with 3 participants and 1 with 6 participants.

**Table 2:** Overview of the activity-oriented questions included in the focus groups

Activity	YP	Adults	Parents	HCPs
Warm-up “rapid-fire” quiz		✓	✓	
Data visualizations	✓	✓	✓	✓
Ideas on sticky notes	✓	✓	✓	
Rating personal experience		✓	✓	
Personas and scenarios	✓			
Prototype screenshots	✓	✓	✓	✓

### Data Analysis

All focus groups were audio recorded and transcribed. Adopting an applied approach [23], thematic analysis [24] and charting [25] were used to search for data patterns within and across the different participant groups. An initial coding frame was developed by 2 researchers (LS and AV), one of whom had not been involved with data collection. This included independent open coding of 4 transcripts each and joint discussion to agree on a comprehensive coding frame. Through constant comparison [26], all data were coded into the coding frame, which was iterated when required and reapplied to the earlier transcripts. The initial coding of the 4 transcripts (representing approximately 35% of the dataset) was cross-checked for reliability; this yielded 93% agreement (κ=0.922, *P*<.001). Once all of the data were organized into the refined coding frame, members of the wider research team (LS, AV, CF, and MC) engaged in detailed discussions, which led to the identification of overarching, interpretative themes. These themes aimed to capture the essence and strength of the participants’ experiences and views; by assigning individual codes to these themes, we were able to maintain a close fit between the data and the more abstract, interpretative themes. Theme-level matrixes [25] were created to compare the nature and distribution of the data across the participant groups.

Questionnaire data were entered into a database and analyzed in SPSS 21 (IBM) by 1 team member (CF).

##  Results

### Overview

Table 3 summarizes the demographic characteristics of the 59 participants. Both the patient and parent samples were predominantly white British, and were mainly male, and female, respectively. In all groups, there was a wide spread of ages, apart from YP, who were all aged 12 or 13 years. The HCP sample included 9 medical staff and 15 non-medical clinical staff, of which 11 were prescribers, 5 were in non-clinical health care roles, and 2 were IT managers. 

Using digital technology was a frequent activity in all participants’ lives. The vast majority of HCPs (27/31, 87%), adults (10/11, 91%), and parents (7/8, 88%) used mobile phones on a daily basis, whereas YP were more likely to use game consoles (5/8, 63%) and tablet computers (5/8, 63%) on a daily basis. Participants mostly used these devices to access the Internet (54/59, 92%), while a majority also used them for apps (39/59, 66%) and some adults, YP, and parents regularly used them for playing games (15/28, 54%).

The qualitative analysis resulted in 5 key themes (see Table 4), 2 of which related to treatment and support for ADHD (ie, complexity of medication decision making and access to diagnosis, treatment, and support), while the other 3 related to the role of RMT (potential of RMT to support people living with ADHD, barriers and limitations to technology, and imagining the ideal app). These themes are described and expanded on below, and are supported by key quotations from the participants. Although all 4 participant groups provided data to support all 5 themes, we highlight any discrepancies in emphasis across participant groups in the theme description.

**Table 3:** Participant demographic information

Participant Group	Total	Gender (female)	Age range (years)	Ethnicity (white British)	Employment status (employed)
Health care professional	31	22	18-64	25	31
Adult	11	7	18-54	10	7
Young person	8	1	12-13	7	0
Parent	9	7	25-54	8	5

**Table 4:** Overview of the analytic interpretative themes and contributing data codes

Analytic interpretative themes	Subthemes	Contributing data codes
Complexity of medication decision making		Personal expectations of medications
		Medication—decision making
		Medication effects
		Confidence in prescribing
		Communication with education professionals
		Questionnaires
		Communication with patients
Access to diagnosis, treatment, and support		Medication—experience of titration
		Experience of diagnosis (of ADHD)
		Communication with health care professionals
Potential of RMT to support living with ADHD	Symptom tracking to improve the quality of clinic appointments	Range of current use of websites
	Supporting greater self-management	Range of current use of other technology
		Anticipated impact—self management
		Anticipated impact—health care consultation
		Acceptability and receptiveness (positive)
		Tracking
		Medication—experience
Barriers and limitations to using RMT	Access to technology	Barriers and limitations (negative)
	Perceived challenges of incorporating RMT into clinical care	
Imagining an ideal app	Organization aid	Content in ideal app
	Coach/supporter/ motivator	
	Reliable, trustworthy and tailored information	
	Monitoring and tracking side effects and symptoms	

### Complexity of Medication Decision Making

**Figure 4:**
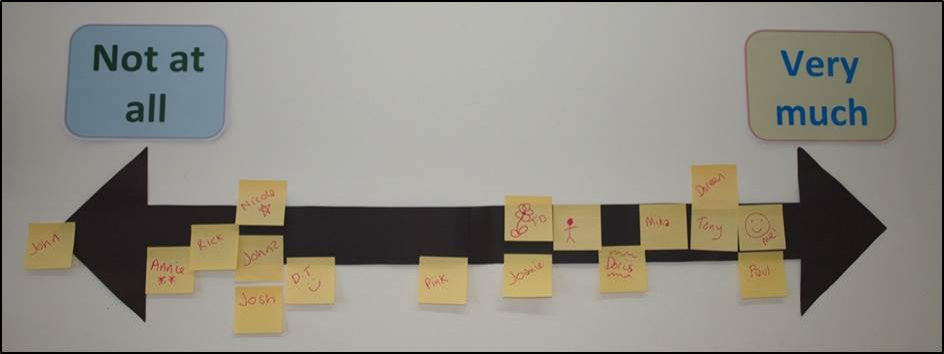
Visualization of adult and parent satisfaction with the titration period. (Note: this is a representation of data and all names are pseudonyms).

Across the sample, experiences of the titration period were mixed. Some participants were content with the speed of the titration and their level of contact with the clinic, while others believed it to be a lengthy period to attain an acceptable level of medication effect (up to 18 months). Very few participants reported that titration had been achieved within the 6-week period recommended by the NICE ADHD Guideline, and most participants reported monthly or less face-to-face contact with the clinic during titration. While few adults were satisfied with this amount of contact, most of the parents were. In the adult and parent groups, a visual scale was used for participants to indicate their satisfaction with the titration process (see Figure 4). This composite visualization (re-created to include all participants’ responses and pseudonyms) shows a broad variation in satisfaction.

HCPs generally reported that while titration may not always meet NICE guideline timescales, their practice was as good as could be delivered within current facilities and resources—more frequent contact was prevented by limited clinic capacity or high caseloads. Weekly monitoring and dose changes were difficult to achieve as prescribers gathered information from a number of sources (such as school reports) to guide their decisions.

You need information from the young person, the parent/carer, and ideally from the teacher, because sometimes the young person doesn’t realize that the medication is beneficial but the parent does.

HCP, Site 1

Despite being optimistic about the effects of the medication when first prescribed, many participants described difficulties in deciding whether to start or continue with it. Adults and parents wanted to achieve an acceptable balance of positive effects and side effects.

It’s a balancing act of getting enough done…You need to say when it is “good enough”. Not getting everything done but weighing up the health issues and side effects.

Adult, Site 1

Because of these experiences, many participants chose when to take (or give their child) medication. HCPs were aware of this and had differing degrees of acceptance of this. Whereas some supported the patient or parent taking control, others were concerned about inconsistent medication taking.

The use of clinical rating scales (or ROMs) to support titration was not consistent across the study sites; where they were used, it was always as an adjunct to the detailed, qualitative information gathered in conversation and written school reports.

You can get a better understanding through a telephone call and you can unpick things more in a conversation, for example, a child is sleeping more than they used to but not as much as the parent would like.

HCP, Site 2

HCPs across all groups raised concerns about the validity and reliability of ROMs, such as whether they are sensitive enough to detect subtle effects. ROMs were also believed to become less informative with repetitive use, open to manipulation, and difficult to interpret when responses differed between respondents (eg, parents, teachers).

### Access to Diagnosis, Treatment, and Support

Despite the primary focus of this study being the use of RMT during the titration period, the focus group discussions were dominated by issues relating to access to diagnosis, treatment, and support, especially for adults and parents. Participants recalled the “*frustrating*” and “*lengthy process*” of getting a diagnosis and that it often took something “*drastic*,” such as a “*breakdown*”, “*meltdown*,” or a number of job losses, before they were taken seriously. Adults specifically mentioned that “*getting someone to listen*” or “*having a doctor that believed in [ADHD]*” was key to their diagnosis, as opposed to being “*fobbed off*,” “*labeled lazy and idle,”* or *“[labeled as] naughty*.” The difficulties in access to diagnosis, treatment, and support appeared to eclipse the experience of medication titration. By the point of diagnosis and commencing medication, adults and parents were so relieved to have access to treatment and support that the time needed to reach the optimum dose of medication was of less importance to them. This experience was supported by comments from some HCPs, as well.

My cases are usually diagnosed in adolescence so they have been waiting for their ADHD to be treated for years, so a few more weeks [for titration] is not a big deal. The important thing is that the process has started.

HCP, Site 1

Although there were some examples of positive experiences, most parents and adults highlighted substantial organizational and logistical issues regarding access to treatment; in particular, they cited long waiting times, brief appointments, lengthy intervals between appointments, inconsistent doctors, unpredictable communication, and frequent cancellations.

Both parent and adult groups expressed a need for further support. One adult specifically reported *“I’m getting down, I’m not coping. I feel like I’ve been left on my own a bit really” [Adult, Site 2].* In addition to support for side effects and symptoms, participants expressed the need for *“reassurance” [Parent, Site 2], “someone there if you have a problem” [Parent, Site 3],* and *“information from someone who “gets it” more than professionals do” [Adult, Site 1].*

### Potential of RMT to Support Living With ADHD

As described above, there was widespread use of Internet-enabled mobile devices, computers, and game consoles across the sample, but their use in relation to ADHD was fairly limited. With the exception of YP, all other groups reported using websites for ADHD information. Parents, adults, and HCPs were cautious about Web information; they wanted to access (or recommend) only trusted or reputable websites. A small number of participants used existing generic mobile phone functions to support daily activities (calendar, reminders, lists, and timers). HCPs described how they sometimes recommended general relaxation and meditation apps, rather than apps specifically designed for people with ADHD.

However, across the whole sample, there were highly positive attitudes toward the potential for RMT to play a greater role in the management of ADHD. Many participants reported that it could improve communication with the clinic; furthermore, participants across all groups saw its potential to (a) improve the quality of the clinic appointments/consultations and (b) support greater patient self-management.

#### Symptom Tracking to Improve the Quality of Clinic Appointments

Parents, adults, and HCPs thought that using RMT would save time in clinic appointments by enabling a quicker review of the patient’s recent history and thereby swifter identification of what matters most to the patient/parent.

This way you can look back over the previous 4 weeks or 3 months and focus on questions such as—“you scored sleep a 2 here, what was happening at the time that made it so unsettled?” It should help parents to be more productive in giving the information we need.

HCP, Site 3

Participants also thought that data collected contemporaneously, rather than retrospectively, would not be biased by patient recall and selective memory.

#### Supporting Greater Self-Management

Participants saw the potential for RMT to provide the ability to easily monitor symptoms, chart them over time, and identify any patterns or unusual behaviors. This would increase people’s knowledge, self-awareness, and understanding of and confidence in dealing with their condition. All of the groups also saw the potential for RMT to offer personalized feedback in response to patient-entered data. This could be used for reassurance, to avoid unnecessary contact with the clinic, and for motivating feedback to reinforce positive behaviors.

It could be a bar with red at the bottom and green at the top and a little person and after each check-up, it moves up or down to show how well you are doing so it gives you the opportunity to realize and change it.

YP, Site 3

Sometimes I think I’m doing well for a few weeks, then I look back and realize I wasn’t. Something to help me accurately monitor that. That in itself would be an aid to the medication… so you can look back and see what you’ve done well and what things you need to concentrate on doing better.

Adult, Site 2

While participants broadly welcomed the concept of RMT, specific feedback on the prototype pointed to some important limitations in this approach. For YP, adults, and parents, the prototype did not allow them to retain and use their own (or the child’s) data, and therefore was unable to realize their vision of its supporting greater self-management described above. For HCPs, the primary issue was that the prototype was perceived as onerous to use and difficult to incorporate into current workflows (for a further description of this, see the “Barriers and limitations to using RMT” section below). They emphasized the need for an RMT that saves clinician time rather than adds to it by requiring additional management outside of the current electronic patient record.

### Barriers and Limitations to Using RMT

The barriers and limitations to using RMT were highlighted in all focus groups, although HCPs foresaw more barriers than did parents, YP, or adults.

#### Access to Technology

Although most participants used Internet-enabled mobile devices, they thought that other people might have had difficulties in physically accessing the technology or having the necessary skills to use it. This included access to mobile devices, especially ones that would support any new app/software (all groups); access to phones when at school (YP); access to wireless Internet in different locations (YP and HCPs); and capability for interoperability with NHS hardware (HCPs).

#### Perceived Challenges of Incorporating RMT Into Clinical Care

RMT was seen as a positive addition to the clinical process only if it *“adds to what’s there already...not if it’s used as an excuse to see people less” [Adult, Site 2].* HCPs were keen on receiving digital information that coincided with patients’ appointments, but noted that *“if you start getting notifications about patients from another area clinic it will take up too much time” [HCP, Site 3]*, and stressed that all of their time spent dealing with digital data be accounted for and appropriately factored into the provider contract. Confidentiality of personal data and privacy was discussed by several groups, as was the need to consider information governance problems and to protect young people from any harms arising from digital tools.

Some HCPs and adults were concerned with providing information digitally to clinics, as it may not be responded to promptly, whereas a phone call would be more likely to elicit a response. Others felt this could be useful information, as long as it was understood that the information might not be seen immediately and that patients should be directed to alternative sources of support when needed.

### Imagining an Ideal App

Participants across all groups were keen to imagine and discuss the functions of their ideal mobile phone app for ADHD. There were similarities across groups regarding the proposed primary functions as well as the preference for personalization, regarding both users’ ability to control settings and to receive tailored responses. YP also wanted any tool intended for daily use to be *“challenging… and funny” [YP, Site 3].* Taken together, the feedback on the prototype RMT and their views on an ideal app points toward a preference for RMT to be controlled by patients, rather than clinics. Four primary functions were identified: organizational aid; a digital coach or mentor; reliable, trustworthy, and tailored information; and symptom or side effect monitoring.

#### Organization Aid

Organization aids were frequently mentioned in all groups, apart from YP. For example, 1 HCP thought that a reminder system would be useful:

The difficulties I come across, [are that] young people are on medication and they tend to run out at the end of the month and their behavior will go sky high, and it will take them a week to get all the medication back into their system. I think it would be really useful if somewhere in the app, say when they’re...near the end [they receive a message saying] “You need to put in a request for repeat prescription.”

HCP, Site 3

Parents supported this idea, especially as YP begin taking more responsibility for managing their ADHD. Adults wanted support with day-to-day tasks: *“something that helps you keep on task and achieve goals...incorporating pre-set plans and lists of tasks” [Adult, Site 2]*. Another discussed the utility of a timing app*:*

To figure out how long it takes you to do certain daily tasks eg, showering. The app could then tell you what time you would need to get up to get everything done [Adult, Site 1].

#### A Coach/Supporter/Motivator

Adults, YP, and HCPs identified potential features that could act as a virtual *“coach” [Adult, Site 1]*, such as providing supportive statements and motivation:

[It] could have some information based on how long the process should take with messages such as “you may not be seeing any improvements yet, but stick with it”... or you could have messages to parents, such as “Derek might be struggling this week”

HCP, Site 3

#### Reliable, Trustworthy, and Tailored Information

All participant groups suggested that the app should provide reliable and precise information, based on both personal experiences and professional knowledge. Information about medications was in particular sought by adults, while parents and HCPs wanted links to reputable sites and resources, including existing apps and support groups; YP wanted information about ADHD books. HCPs suggested that an app could give proactive advice about behavior, sleep, and diet. They also felt advice should be targeted with frequently asked questions and specific, tailored responses, such as *“I’m feeling dizzy—[the] advice would be to go to see your GP” [HCP, Site 4]*.

#### Monitoring and Tracking Side Effects and Symptoms

There was strong support across all groups to track information visually, such as using a graph to illustrate changes in symptoms, side effects, and behavior over time. YP suggested that monitoring mood swings and side effects might be useful. HCPs wanted the ability to monitor medication compliance and side effects, as well as tracking behaviors such as eating, sleeping, and drug and alcohol use during titration. The usefulness of being able to link this information with life events was also identified:

Graphs would be useful for example for... patients who stop taking meds but parents and teachers say they have improved. It might help to have the parent and teacher graphs to see.

HPC, Site 4

Adults wanted the mode of recording information to be tailored to individual preferences, for example, using smiley faces to record mood, using the voice recorder, and linking with other apps such as work calendars. HCPs felt it would be useful if school staff could provide feedback through the app. Parents were happy for YP to enter their own data when they were mature enough, but also wanted to provide feedback and report how they themselves are doing.

## Discussion

### Principal Results

The participants in this study reflect current trends in technology use [12,13], particularly with regard to their high use of mobile technologies (eg, Internet-enabled mobile phones and tablets). While few currently used these devices in relation to ADHD (ie, patients or HCPs), we found widespread support for augmenting ADHD treatment and support with RMT, eHealth, and mHealth apps. However, 2 important findings from this study indicate that the purpose and functions of the initial prototype did not align with participants’ priorities.

First, while our initial interest in conducting this study was to explore how RMT could improve titration management, and even though our findings support audit work suggesting that the NICE guidelines are not currently met in NHS services (personal communication (unpublished audit) by Hall, 2015), few participants identified this as an area of high unmet need. The exploratory nature of the study enabled new ideas to emerge that moved us beyond titration. Through participants’ detailed accounts, we identified 3 clear phases of living with ADHD: initial assessment and diagnosis; starting treatment (including medication titration); and ongoing support and management. The group discussions with adults, YP, and parents were dominated by their experience of the first phase—namely, trying to access services and getting a formal diagnosis, as described in our second theme (access to diagnosis, treatment, and support). This adds to previous evidence of this being an area of high unmet need [2]. Once diagnosed, the experience of relief and optimism was similar to that reported in other studies [27], as was their recognition of the long-term implications of ADHD [28], the need for ongoing support in living with ADHD, and the limitations of medication [27].

Second, participants identified that RMT’s greatest potential was in improving ongoing support and management of ADHD. The use of RMT to improve ADHD by continuing the health care interventions between appointments has previously been recognized [14]. In this study, 2 key advantages were highlighted—tracking to improve the quality of clinic appointments and support for greater self-management.

While there have been calls for increased use of ROMs and clinical measures to enhance clinical practice [29], there are several barriers to widespread adoption [30]. Some of these barriers were identified in this study—specifically, increased burden on clinics, questions about the clinical utility of the data, and the need for information from a range of sources, particularly regarding medication-decision making. It has been argued that technology may make ROMs more useful by providing data in a form that is timely and accessible by patients, families, and clinicians [31]. Our findings support those of Hall et al. [11], who found that regular monitoring using electronic ROMs allowed for tracking progress and facilitating communication and engagement. As identified by participants in this study, using mobile phone/tablet apps for completing ROMs has the potential to save time for both clinicians and patients [32] and may provide more accurate data than paper-based questionnaires [33]; however, some academics caution against simple translation of paper-based questionnaires to digital formats without further validation [34].

Participants in this study demonstrated a preference for an RMT system that supports greater self-management—namely, one that is patient-owned and controlled, such that patients/parents can choose when and how to use it and specifically when to share the information with their clinician. The widespread adoption of mobile phones and tablet computers in the population makes them advantageous over other digital devices as they are very portable and frequently in the owner’s possession throughout the day [15]. These devices afford the opportunity for the use of low-cost apps that can support clinical management at any time of day or in any location [35] through provision of trusted information, real-time monitoring and symptom tracking, prompts and medication reminders, personalized behavioral support, and communication with health care services. However, the value of apps to the health care system will only be fully realized once the data generated from them is shared between patients, carers, and clinicians to improve their efficiency and quality of care.

Although there are some ADHD-specific mobile phone apps available, we are not aware of any evaluations or evidence of their clinical benefit. Research has shown that using generic mobile phone features such as calendars, task lists, and notes, with the support of a human online coach, can be effective in managing ADHD symptoms [36]. Extending this approach with a bespoke ADHD tool that incorporates the 4 key features identified in this study (organizational aid, virtual coach, reliable and tailored information, and monitoring and feedback) is proposed as the next step for research. This tool also needs to include positive reinforcement such as rewards for completion and game-like features (a process called “gamification”) to enhance user engagement [14], which could meet YP requirements for any tools to be fun and challenging. In addition to rating symptoms and treatment progress monitoring, an RMT system could complement therapy, psychoeducation, support, and advice [37].

Our study has demonstrated the vital importance of developing systems in collaboration with the end users. The next stage of research and development for RMT for ADHD will be to adopt well-established user-centered approaches with rapid, iterative cycles of requirements elicitation, design, testing, and redesign [38]. The findings of this study provide a strong foundation to commence this stage, and will also help with testing the efficacy of the RMT during its development; this is a crucial part of beginning to build evidence for the clinical effectiveness of any new RMT.

Greater patient activation, including self-management and self-monitoring, is a goal for many long-term conditions, not only ADHD. The primary findings from this study—that RMT has a place throughout the patient’s journey (not only targeted at a single stage) and the overwhelming preference for patient-controlled, rather than clinic-controlled systems—may also apply across other neurodevelopmental and mental health disorders. Our use of the HCP sample in this study adds support to this point, as they came from CAMHS and pediatric services where they would treat a range of different conditions. However, it will be important to test these assumptions with other populations; in particular, the detailed user requirements for an ADHD-focused RMT may not transfer directly to other conditions and populations.

### Strengths and Limitations

By engaging with a range of participants, we have explored the commonalities and differences across the key stakeholder groups. We found universal support for technology innovation across these groups and identified their preferences for how this could be implemented. Diversity with respect to gender, age, and ethnicity across our sample was limited, meaning that we may have missed some important perspectives. However, it should be noted that the ethnic profile of our sample reflects previous findings of studies of access to care, where white children were twice as likely as access services than were children from other ethnic groups [39].

By using a defined prototype as a discussion vehicle for RMT within a defined clinical condition, we may have narrowed the scope of the discussion and the ideas of what would be useful or possible. For example, while access to diagnosis was identified as the most significant unmet need, participants’ aspirations for technological support focused on treatment and support. However, many of the ideas proposed diverged from the specific approach taken with the prototype, suggesting that participants had clear ideas for when and how technology would be helpful to them.

### Conclusions

The findings from this study strongly suggest that YP, parents, and adults with ADHD are looking for a more personalized, responsive approach to ADHD treatment and support in the long term. Patients and their families want more targeted information at a time they need it most, to have control over interventions including medication, to have facilities to record and monitor personal and sensitive information about themselves, and to have support in developing personal strategies that fit with their lives. This study of patients’, parents’, and HCPs’ views of using RMT for ADHD leads us to the conclusion that a technology-based personalized approach for living with ADHD, driven by user requirements, is required. However, implementing RMT requires a key change in the philosophy of health care from routinized clinic-centered care to personalized patient-centered care.

## Appendix

Multimedia Appendix 1 Example focus group schedule: Parents.

## Abbreviations

ADHD: Attention deficit hyperactivity disorder
CAMHS: Child and adolescent mental health services
HCP: Health care professionals
NHS: National Health Service
NICE: National Institute for Health and Care Excellence
NIHR: National Institute for Health Research
ROM: Routine outcome measure
RMT: Remote monitoring technology
UK: United Kingdom
YP: Young people
