# Supplementary material for: Developing mHealth Remote Monitoring Technology for Attention Deficit Hyperactivity Disorder: A Qualitative Study Eliciting User Priorities and Needs
Source: JMIR Mhealth Uhealth. 2016 Mar 23;4(1):e31. doi: 10.2196/mhealth.5009 (PMC4823590; doi:10.2196/mhealth.5009)
Supplement: Multimedia Appendix 1 [file mhealth_v4i1e31_app1.pdf]

RMT for ADHD study
Focus group 5 (Parent)
9th July 2014
10-12pm Parent support group

Moderator – Lucy
Observer – Zoe

On arrival

1) Name badges – name want to be known by in the group
2) Check have read the participant information sheet and whether have any questions about the study
3) Consent forms – 2 copies each
4) Complete demographic questionnaires

At start of the group

1) Ways of working – point out poster and ask if want to add anything
    - Respect privacy
    - It's OK to disagree but it's not OK to be disagreeable
    - One person speaking at a time
    - Try not to interrupt
    - Speak up and clearly for the recording
2) Explain people's roles
    - LS – discussion questions, process and timing
    - ZY - questionnaires, tape recording and notes (general flavour of discussion & strong/divergent views, how questions are working)
3) Remind participants
    - they can leave at any point if they change their mind about taking part
    - our agreed finish time

General probing questions (crib for moderator)

- Does anybody else have anything to add to that?
- [name of person] raised the point that …, what do the rest of you feel about that?
- I noticed somebody frowning that that point, does someone have another view on that issue?
- I'm not sure I understood that comment, could you tell me more about that?
- We haven't heard from [area of table, name of participant] yet, have you got anything to add to that?

<u>Section 1. Technology for Health</u>

**1**	**Quiz about technology use**
Introductory<br><br>*10 mins*	Shout out your answer to the following questions:<br>a) How many Britons access the internet regularly?<br>b) How many Britons own a smartphone?<br>c) How many Britons own a tablet?<br>d) What is the most frequent way adults keep in touch?<br><br>Answer yes/no by sticking a sticky dot on the poster:<br>e) Would you like to monitor a chronic condition using a mobile app? Answer = 60%<br>f) Do you search for health information online? Answer = 70%<br>g) Would you like to view your medical records online? Answer = 80%<br>h) Would you use an online GP appointment booking service? Answer = 90%<br>i) Would you use a service letting you ask a clinician a question? Answer = 90%
**2**	**Can you give an example of how you use technology (for health)?**
Key<br><br>*10 mins*	*Probes*<br>• *apps, websites etc.*<br>• *what do you use it for? information seeking, diagnosis, NHS choices*<br>• *How did you choose what to use?*<br>• *What do you like about them?*<br>• *What benefits do they bring?*<br>• *If anyone doesn't use technology, why not?*<br><br>Notes: note down specific apps mentioned

<u>Section 2. Experiences of Medication Titration</u>

**2**<br><br>*Key*<br><br>*10 mins*	Evidence suggests that patients reach the best medication dose quickly and without too many side effects when they have regular contact with their doctor and a dose schedule that is individual to that person.<br><br>ADHD guidelines state that contact should be every week and at least at every dose change. We want to learn more from you about your experiences from starting medication to reaching the right dose.<br><br>**Poster 1: What did you expect when your child started medication?**<br>(Write 3 bullet points on a post-it note and stick to poster)

Discuss responses

Probes

- *What type of contact did you have with the clinic?*
- *Was there enough contact?*
- *Were you happy with the types of contact?*
- *Did your son/daughter experience side effects?*
- *How were these dealt with?*

3 **Poster 2: How satisfied were you with the process when your child started medication?**

Key

5 mins

(Write your name on post-it note and stick it to the scale: Very satisfied to not satisfied at all)

4 **Poster 3: How could this process have been improved?** Check Poster 2 to see if this question needed

Key

5 mins

Option 1: Build Poster 3 together through discussion**,** noting down responses on flip-chart.

Option 2: Write answers on post-it notes and stick to flip-chart (if discussion is lacking)

Probes

- *I can see that you weren't very satisfied, why is that? What would have been helpful at this time?*
- *What made it a good experience?*
- *In what ways would these suggestions have helped you? Would they have benefitted anyone else (family, siblings, teacher etc)?*

********* Link sections 1 and 2 - use of tech to support *********
people when taking medication

<u>Section 3. Remote monitoring for effects and side effects</u>

5 **Introduce the prototype monitoring system**

Key

10-15 mins

In some places when people visit the ADHD clinic they are asked to fill in questionnaires (usually on paper) about their child's symptoms (is your child feeling restless etc) and side effects (is your child feeling poorly etc) while they are waiting to see their doctor. The doctor then talks to the patient about the answers to these questionnaires in the appointment.

We are interested in using smartphones to collect this information before people get to the clinic. Patients/parents will receive a text message reminder to fill in 2 questionnaires, one about how you're child is getting on at school and home (are they able to concentrate better etc) and one about your child's medication and how it's making them feel (are they sleeping more or less? Are they eating more or less? Are there any side effects?).

Responses would be sent straight to the doctor (or the person who gives your child their medication) who can then look at these before you arrive at clinic.

Doctors may be able to change how much medication your child takes at this point or they may decide to change the date of the clinic appointment depending on what the information says.

Pass around screenshots of the text messages and online questionnaires

What do you think?

Probes –

- *Would you be interested in this?*
- *What do you think could be the advantages of this system?*
- *How would it fit in with your daily activities?*
- *What are the barriers to this?*
- *How would you feel about reminders?*
- *What if you kept getting reminders at work or when you're busy?*

6 **In what ways might the clinic respond to the information you send?**

Key

5 - 10 mins

Probes

- *If you/your child reported a side effect, what would you want the clinic to do about it?*
- *How would you feel about positive/stable reports from the tool?*
- *If you had stable reports, would you like your child to have less frequent appointments at the clinic?*
- *How would you feel about this?*

7 **With this system, it would be possible to create a report of your responses over time – would you be interested in this?**

Key

5 - 10 mins

Probes

- *How would you like this to look?*
- *Options e.g. monthly report vs. as and when you wish to view?*
- *Would you like to share your answers/ reports? If so who with? partner, teacher, parent, support worker*

Section 4. Aspirations for using technology to manage ADHD

8 **What do you think about using technology for ADHD?**

Key

5 mins

Probes
- *Positives?*
- *Negatives?*
- *information sharing, access to technology, reminders*

9 **Imagine you could design the perfect app, what would it include?**

Key

5 mins

Probes (taken from the user workshop: self-help, appointment schedule, medication reminders, task reminders, user forum/shared community, information/advice/guidance, help button, verbal notes, ability to defer/snooze reminders, links to other websites, a measure of improvement in work/social life- not just symptoms

Closing the session
- Draw discussion to a close
- Ask if there are any other points anybody wants to raise
- Thank everyone
- Sort out vouchers
- Deal with any questions
- show participants out.

De-briefing points to discuss
- Atmosphere of the group
- Main topics discussed in group
- Topics raised spontaneously or after questioning
- Interaction between participants
- Any areas of conflict/disagreement
- Questions/topic areas that worked and any that didn't
- Suggested changes to schedule
